# Pediatric Caregiver Attitudes Toward Email Communication: Survey in an Urban Primary Care Setting

**DOI:** 10.2196/jmir.2738

**Published:** 2013-10-23

**Authors:** Robert Arthur Dudas, Michael Crocetti

**Affiliations:** ^1^Johns Hopkins Bayview Medical CenterDepartment of PediatricsJohns Hopkins University School of MedicineBaltimore, MDUnited States; ^2^Johns Hopkins Community PhysiciansDepartment of PediatricsJohns Hopkins University School of MedicineBaltimore, MDUnited States

**Keywords:** electronic mail, email, primary health care, communication, health care disparities, pediatrics

## Abstract

**Background:**

Overall usage of email communication between patients and physicians continues to increase, due in part to expanding the adoption of electronic health records and patient portals. Unequal access and acceptance of these technologies has the potential to exacerbate disparities in care. Little is known about the attitudes of pediatric caregivers with regard to their acceptance of email as a means to communicate with their health care providers.

**Objective:**

We conducted a survey to assess pediatric caregiver access to and attitudes toward the use of electronic communication modalities to communicate with health care providers in an urban pediatric primary care clinic.

**Methods:**

Participants were pediatric caregivers recruited from an urban pediatric primary care clinic in Baltimore, Maryland, who completed a 35-item questionnaire in this cross-sectional study.

**Results:**

Of the 229 caregivers who completed the survey (91.2% response rate), 171 (74.6%) reported that they use email to communicate with others. Of the email users, 145 respondents (86.3%) stated that they would like to email doctors, although only 18 (10.7%) actually do so. Among email users, African-American caregivers were much less likely to support the expanded use of email communication with health care providers (adjusted OR 0.34, 95% CI 0.14-0.82) as were those with annual incomes less than US $30,000 (adjusted OR 0.26, 95% CI 0.09-0.74).

**Conclusions:**

Caregivers of children have access to email and many would be interested in communicating with health care providers. However, African-Americans and those in lower socioeconomic groups were much less likely to have positive attitudes toward email.

## Introduction

It has been more than 10 years since the Institute of Medicine recommended “patients should receive care whenever they need it and in many forms, not just face-to-face visits” and “access to care should be provided over the Internet” [1]. Although electronic methods have the potential to increase communications and the quality and efficiency of care, most research has focused on adult populations [2] and the evidence base is inadequate to assess the effect of email for clinical communication between caregivers and health care professionals [3]. Nevertheless, a study conducted with parents who used email to communicate with their child’s pediatrician found that 98% of parents were very satisfied with their experience [4], whereas another study suggested that email enabled physicians to answer medical questions in less time compared with telephone messaging [5]. Surveys from the United States have revealed wide variability in email practices with 16% of physicians using email to communicate with patients in a survey of primary care practitioners to as many as 72% in a large outpatients’ department [6,7]. A younger patient population may correlate with increased usage as the majority (79%) of doctors at a student health center in Finland reported email use with their patients [8].

Overall usage of email communication between patient and physician continues to increase, but because of expanding usage of electronic health records and patient portals, there is a concern that unequal access to these technologies could exacerbate disparities in care. Medicaid patients and black patients were less likely to have access to email in pilot studies involving an email intervention [9]. Others have shown that nonwhite or low socioeconomic status patients were less likely to use email in a Web-based secure portal despite access to the necessary technology [10].

We hypothesize that parents bringing their children to a pediatric primary care clinic have access to the Internet and email and would be interested in communicating with their health care providers by these modalities. The aim of this study is to document pediatric caregiver attitudes toward and access to these technologies in an urban pediatric primary care clinic.

## Methods

### Overview

We conducted a cross-sectional survey involving a convenience sample of 300 caregiver-child dyads with children aged from birth to 21 years presenting for care at an urban pediatric primary care clinic in Baltimore, Maryland. The Institutional Review Board of Johns Hopkins University approved this study.

### Study Setting and Participants

This study was undertaken at a community teaching hospital affiliated with a major academic center. The primary care clinic was staffed by 6 pediatric providers and 15 pediatric residents (3 residents each afternoon for continuity clinic).The pediatric clinic has an annual pediatric volume of approximately 10,000.

To be eligible, pediatric patients accompanied by their caregiver had to present to the clinic between November 2010 and January 2011 during clinic hours of 09:00-17:00. Patients were excluded if they were non–English speaking because we lacked the resources to interview them.

### Survey Instrument

We developed a questionnaire based upon existing literature [4,11,12] that was piloted on a group of 10 caregivers before study initiation to identify ambiguity. Changes were made to clarify wording before study initiation based upon feedback. We used a final 35-item paper-based questionnaire that included multiple selection and 5-point scale questions. Survey domains included (1) demographic information, including caregiver and child age, sex, race, education, annual family income, and insurance type; (2) caregiver email usage patterns; and (3) caregiver attitudes toward email.

### Study Protocol

A 35-item survey instrument was distributed to 300 consecutive English-speaking caregivers presenting to the clinic. The survey was distributed to the caregiver by a registrar during the check-in process. The survey was either returned or self-administered and collected at the conclusion of the health care encounter. No incentives were offered to complete the questionnaire, which took approximately 10 minutes to complete.

### Statistical Analysis

Sample size calculations were based upon the determination of the proportion of email users in this cross-sectional study. It was predicted that approximately 80% of the surveyed population would be email users. We assumed a 95% level of confidence and set our precision at .05 to yield a sample size of 246. Data analysis was performed with the use of Stata version 9.2 (StataCorp LP, College Station, TX, USA). Frequencies and simple means were calculated for each variable, where appropriate. For items utilizing a 5-point scale, we considered both “strongly agree” and “agree” as agreement with that item. Unadjusted and adjusted logistic regression was used to assess associations between demographic variables and binary attitudinal variables. The amount of missing data for study variables of interest was minimal with an average missing data rate per variable of 0.8% and the largest missing data occurring for the insurance variable at 6.4%. In the regression analysis, we used listwise deletion to account for missing data. Results are reported as odds ratios (OR) and adjusted odds ratios (adjusted OR) with 95% confidence intervals (CI). A *P* value less than .05 was considered significant.

## Results

**Figure 1:**
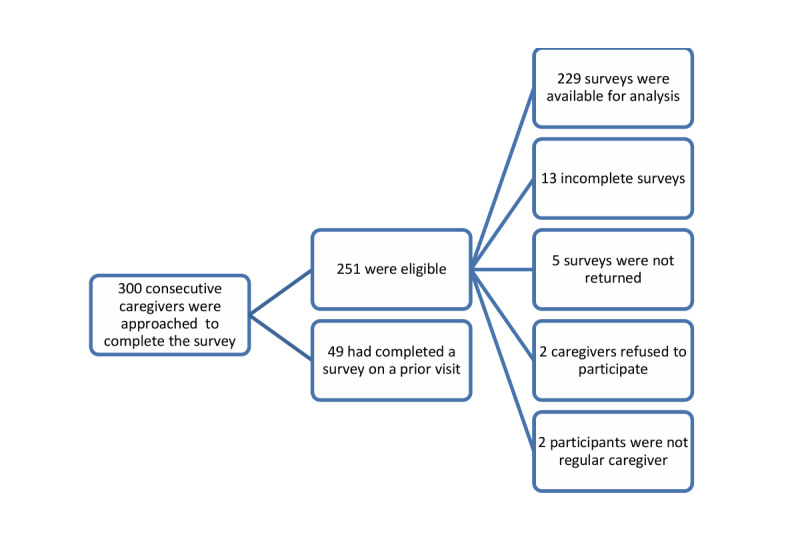
Survey response flow diagram.

From 300 consecutive English-speaking caregivers, 229 surveys were available for analysis (Figure 1).

Of the 229 participants, 171 (74.6%) stated that they use email to communicate with others. Table 1 reveals the demographic characteristics of the caregivers based on email usage. Most respondents were mothers (181/229, 79.0%) with an average age of 33.6 years (SD 10). Caregivers who used email were more likely to have a college or greater education (*P*=.003), higher income (*P*<.001), and commercial insurance (*P*=.002) (Table 1).

Of those who did use email, 86.3% (145/168) reported that they would like to communicate with their provider by this method, although only 10.7% (18/168) reported doing so (Table 2).

A large percentage of respondents that use email agree or strongly agree that more doctors should offer email communication to their patients (135/171, 78.9%) (Table 3). Many also stated that email would be a good way to make an appointment (130/170, 76.4%), increase contact with their child’s provider (131/170, 77.0%), and improve communication with their provider (125/170, 73.5%).

Email users were asked to select their preferred method to receive test and x-ray results; they were evenly split between email (40.2%, 68/169) and phone (40.2%, 68/169) as preferred method with a minority preferring regular mail (16.6%, 28/169) or text messaging (2.9%, 5/169). Additionally, most caregivers felt that email was an appropriate modality to discuss many of the conditions commonly encountered in pediatric practice, including cold symptoms and sleep and weight issues, although they were slightly less comfortable discussing behavior and development issues by email (Table 4).

In the adjusted logistic regression model, African-American caregivers were less likely to agree with the following statements: more providers should offer email (adjusted OR 0.34, 95% CI 0.14-0.82), email would increase contact with the provider (adjusted OR 0.41, 95% CI 0.19-0.91), email with the provider would be satisfying (adjusted OR 0.32, 95% CI 0.14-0.75), and email would be an easy way to make an appointment (adjusted OR 0.4, 95% CI 0.18-0.88) (Table 5).

**Table 1:** Caregiver demographics by email usage (N=229).

Variable		Do you ever use email to communicate with others?		*P* value
		Yes (n=171)	No (n=58)	
Mothers surveyed, n (%)		140 (82.3)^a^	41 (70.6)	.06
Age (years), mean (SD)		33.3 (9)	34.1 (12)	.51
**Race, n (%)**				
	African-American	74 (43.2)	31 (53.4)	.22
	Caucasian	80 (47.3)	25 (43.1)	.65
	Other	17 (9.9)	2 (3.4)	.17
**Education, n (%)**				
	< High school	13 (7.6)^a^	10 (18.1)^b^	.04
	High school (GED)	72 (42.3)^a^	37 (67.3)^b^	.44
	College or greater	86 (50.6) ^a^	8 (14.5) ^b^	<.001
**Income (US $), n (%)**				
	≤20,000	34 (20.0)^a^	28 (50.9)^b^	<.001
	20,001 - 40,000	44 (25.9)^a^	9 (16.4)^b^	.20
	> 40,000	59 (34.7)^a^	1 (1.8)^b^	<.001
	Don’t know/refuse	33 (19.4)^a^	17 (30.9)^b^	.09
**Health insurance, n (%)**				
	Commercial/private	90 (56.2)^c^	18 (32.7)^b^	.003
	Medicaid	61 (38.1)^c^	27 (49.1)^b^	.16
	None	1 (0.6)^c^	0 (0.0)^b^	.99
	Don’t know/refuse	8 (5.0)^c^	10 (18.2)^b^	.004

^a^Percentages calculated based on 170 responses.

^b^Percentages calculated based on 55 responses.

^c^Percentages calculated based on 160 responses.

**Table 2:** Practices of email users.

Variable		Frequency, n (%)
**How often do you check email?** ^a^		
	Daily or more	102 (60.0)
	Few times weekly	35 (20.6)
	Weekly or less	33 (19.4)
Would like to email child’s doctor (yes)^b^		145 (86.3)
Communicates currently with child’s doctor by email (yes)^b^		18 (10.7)

^a^Percentages based 170 responses.

^b^Percentages based on 168 responses.

**Table 3:** Email users attitudes toward email communication with doctors (n=171).

Variable	Frequency of agreement, n (%)
More doctors should offer email communication	135 (78.9)
Email would increase contact with my child’s doctor^a^	131 (77.1)
Email would distance us from my child’s doctor	19 (11.1)
Email would improve communication with my child’s doctor^a^	125 (73.5)
Email with my child’s doctor would be satisfying	131 (76.6)
Email would be a good/easy way to make appointment^a^	130 (76.5)
Email hackers are a worry^a^	58 (34.1)

^a^Percentage calculated based on 170 responses.

**Table 4:** Email communication to discuss common pediatric conditions (N=171).

Pediatric condition	Agreement, n (%)
Cold symptoms	141 (82.5)
Earache	139 (81.3)
Sleep	136 (79.5)
Weight issues	136 (79.5)
Fever	135 (78.9)
Constipation	133 (77.8)
Diarrhea	133 (77.8)
Vomiting	129 (75.4)
Feeding/diet	134 (78.4)
Safety topics^a^	133 (78.2)
Pink eye	131 (76.7)
Toilet training^b^	129 (76.3)
Immunizations	125 (73.1)
Colic^b^	121 (71.6)
Behavior	121 (70.8)
Development^a^	120 (70.6)

^a^Percentages calculated based on 170 responses.

^b^Percentages calculated based on 169 responses.

**Table 5:** Logistic regression of email users’ attitudes as a function of age, education level, insurance type, family income, and race.

Covariate (n)		Agreement	
		OR (95% CI)	Adjusted^a^ OR (95% CI)
**More providers should offer email (171)**			
	Caregiver age	1.05 (1.00-1.09)	1.00 (0.94-1.08)
	Completed > high school	3.45 (1.54-7.72)	2.28 (0.88-5.91)
	Medicaid insurance	0.47 (0.22-1.02)	0.75 (0.30-1.9)
	Income ≤ US $30,000	0.17 (0.07-0.43)	0.26 (0.09-0.74)
	African-American race	0.40 (0.19-0.85)	0.34 (0.14-0.82)
**Email would increase contact with provider (170)**			
	Caregiver age	1.02 (0.98-1.06)	1.02 (0.96-1.09)
	Completed > high school	1.92 (0.92-3.99)	1.33 (0.56-3.17)
	Medicaid insurance	0.64 (0.30-1.35)	0.73 (0.31-1.72)
	Income ≤ US $30,000	0.43 (0.20-0.92)	0.59 (0.24-1.45)
	African-American race	0.38 (0.18-0.79)	0.41 (0.19-0.91)
**Email would distance us from provider (171)**			
	Caregiver age	0.95 (0.89-1.01)	0.95 (0.87-1.05)
	Completed > high school	0.40 (0.15-1.12)	0.49 (0.13-1.82)
	Medicaid insurance	2.16 (0.79-5.9)	1.96 (0.56-6.91)
	Income ≤ US $30,000	3.65 (1.15-11.51)	1.51 (0.39-5.88)
	African-American race	1.53 (0.59-3.98)	1.40 (0.46-4.27)
**Email would improve communication with provider (170)**			
	Caregiver age	1.03 (0.99-1.07)	1.05 (0.99-1.12)
	Completed > high school	1.82 (0.91-3.63)	1.37 (0.59-3.20)
	Medicaid insurance	0.83 (0.41-1.68)	1.10 (0.48-2.52)
	Income ≤ US $30,000	0.48 (0.24-0.99)	0.57 (0.24-1.38)
	African-American race	0.45 (0.23-0.90)	0.49 (0.23-1.04)
**Email with provider would be satisfying (171)**			
	Caregiver age	1.01 (0.97-1.05)	1.00 (0.95-1.07)
	Completed > high school	2.34 (1.12-4.88)	1.67 (0.68-4.12)
	Medicaid insurance	0.55 (0.26-1.17)	0.79 (0.33-1.91)
	Income ≤ $30,000	0.21 (0.09-0.49)	0.25 (0.09-0.66)
	African-American race	0.31 (0.15-0.65)	0.32 (0.14-0.75)
**Email would be easy way to make appointment (170)**			
	Caregiver age	1.02 (0.98-1.06)	0.99 (0.94-1.06)
	Completed > high school	1.78 (0.86-3.65)	1.90 (0.79-4.58)
	Medicaid insurance	0.91 (0.44-1.90)	1.39 (0.59-3.29)
	Income ≤ US $30,000	0.47 (0.23-0.99)	0.53 (0.21-1.33)
	African-American race	0.41 (0.20-0.85)	0.40 (0.18-0.88)
**Email hackers are a worry (170)**			
	Caregiver age	0.97 (0.94-1.01)	0.98 (0.93-1.04)
	Completed > high school	0.53 (0.28-1.01)	0.83 (0.38-1.81)
	Medicaid insurance	1.78 (0.92-3.44)	1.35 (0.62-2.95)
	Income ≤ US $30,000	3.06 (1.56-6.02)	2.21 (0.97-5.02)
	African-American race	1.60 (0.85-3.02)	1.34 (0.65-2.73)

^a^Adjusted for all other covariates in a multiple logistic regression model.

## Discussion

### Principal Results

Most caregivers in our urban population have access to email and are interested in communicating with their child’s providers by this method, although only 11% currently communicate with their provider via email. Overall, attitudes toward email were favorable with three-quarters of email users reporting that email would improve communication with their provider and be satisfying. Despite the generally favorable disposition toward email, most caregivers preferred not to receive test or x-ray results by email. This may be related to the finding that 34% of caregivers remain concerned about security issues surrounding email, which suggests that there are content areas that caregivers feel less comfortable discussing via email. Regarding discussing specific pediatric conditions commonly encountered in primary care, there was much greater acceptance of such topics with no notable differences among the types of conditions.

### Comparison With Prior Work

Our study suggests that caregivers with higher education, higher income, and commercial insurance were more likely to use email to communicate. Furthermore, of those caregivers who did report using email, African-American caregivers and those with incomes less than US $30,000 were less likely to have favorable views toward email as a tool to communicate with their health care providers. Although others have suggested that the digital divide is a function of decreased access to email for these groups [9], our data offer further evidence that disparities persist across racial/ethnic and socioeconomic groups even after controlling for access to email and Internet [10]. There may be factors related to the quality of Internet connection or perhaps cultural preferences pertaining to privacy or other factors as yet not determined. Increased emphasis on the meaningful use of electronic health records has led health care systems to develop patient portals that offer access to health information and frequently allow for 2-way secure messaging between patients and providers. However, recent studies are reporting disparities in adult and pediatric patient portal use along racial and socioeconomic lines [13-15]. A recent review on patient-provider email suggests that email has tremendous potential to improve health care communication between patients and providers and should lead to improved satisfaction and quality of care [16]. Yet, the evidence base to assess the effect of email on clinical communication remains limited and of poor quality [3]. Many questions concerning access, acceptance, privacy, and security issues remain unanswered and established national guidelines are currently lacking.

### Limitations

Study limitations include the small sample size and cross-sectional design. However, we surveyed consecutive caregivers and our response rate was high. Other limitations include bias from our convenience sample at a single urban clinic limiting the ability to generalize our results to other populations. Also, we did not make a distinction between personal and professional email usage (or secure patient portals) which could have implications upon caregiver attitudes toward privacy concerns. Lastly, our survey instrument lacks formal testing for reliability and validity.

Further research should continue to closely monitor for exacerbations of existing disparities in pediatrics as the medical community further embraces email and other electronic data communication methods. Text messaging may serve as another alternative communication modality as it too has been shown to be generally accepted by parents [17]. Investigators should help determine which communication modalities are best suited for conveying specific information such as test results, or providing information about medical conditions while taking into consideration the inherent ethical and privacy concerns raised by all forms of communication [18,19].

### Conclusions

Caregivers of children in an urban pediatric primary care practice have access to email and would be interested in communicating with health care providers by this method. African-American caregivers and those in lower socioeconomic groups hold less favorable views toward email communication; thus, the use of email may exacerbate existing disparities in health care delivery. Future studies should examine the reasons for these attitudinal differences.

## Multimedia Appendix 1

Survey instrument.
