# Supplementary material for: Pediatric Caregiver Attitudes Toward Email Communication: Survey in an Urban Primary Care Setting
Source: J Med Internet Res. 2013 Oct 23;15(10):e228. doi: 10.2196/jmir.2738 (PMC3806434; doi:10.2196/jmir.2738)
Supplement: Supplementary file 1 [file jmir_v15i10e228_app1.pdf]

This is a research study. Your participation is voluntary and will not affect the care your child receives today or in the future. Completing this survey will serve as your consent to participate in this study. Information gathered in this survey will be shared among the study team and may lead to a scientific article. Neither your name nor your child's name will appear on this survey.

# Email Communication Questionnaire

Have you already completed this questionnaire? Yes ____ No ____

(If YES, please stop and hand form in to the front desk)

(If NO, please continue)

Are you one of the main caregivers for the child being seen today and can you answer questions about the child's health? Yes _____ No _____

(If NO, please stop and hand form in to the front desk)

(If YES, please continue)

We would like to ask a few questions for statistical purposes

1. What is your relation to the child being seen today?

   Mother _____ Father _____ Grandparent _____ Other _____

2. What is your age? _____ yrs

3. What is the age of the child or children being seen today?

   Child #1 _____ months **or** _____ years Child #2 _____ months **or** _____ years

4. How would you best describe your race (select one)?

White _____ Black or African-American _____ American Indian _____ Asian _____

Hispanic or Latino _____ Other _____

5. What was the last grade you completed in school?

Elementary (grade 1 – 5) _____ Middle (grade 6 – 8) _____ High School/GED _____

College _____ More than college _____

6. Would you say that your total yearly income of your household is:

Less than or equal to $10,000 _____ $10,001 - $20,000 _____

$20,001 – $30,000 _____ $30,001 – $40,000 _____Greater than $40,000 _____

Don't know _____ Refuse _____

7. What type of health insurance does the child being seen today have?

Medicaid/MCO _____ HMO/PPO _____Private (Blue Cross, EHP) _____

None _____Don't know _____ Refuse _____

8. Do you ever use e-mail to communicate with others? Yes _____ No _____

This is a research study. Your participation is voluntary and will not affect the care your child receives today or in the future. Completing this survey will serve as your consent to participate in this study. Information gathered in this survey will be shared among the study team and may lead to a scientific article. Neither your name nor your child's name will appear on this survey.

(If NO, please stop and hand form in to the front desk)

(If YES, please go on to item 9)

We would like to ask a few questions about your use of e-mail

9. How often do you check your e-mail?

More than once daily _____ Daily _____Few times per week _____Weekly _____

Less than weekly _____

10. Would you like to be able to communicate with your child's doctor by e-mail?

Yes _____No _____

11. Do you already communicate with your child's doctor by email? Yes _____

No _____

12. More doctors should offer e-mail

Strongly agree ___ Agree ___ Neutral ___ Disagree ___ Strongly disagree ___

13. E-mail would increase contact with my child's doctor

Strongly agree ___ Agree ___ Neutral ___ Disagree ___ Strongly disagree ___

14. E-mail would distance me from my child's doctor

Strongly agree ___ Agree ___ Neutral ___ Disagree ___ Strongly disagree ___

15. E-mail with my child's doctor would be satisfying

Strongly agree ___ Agree ___ Neutral ___ Disagree ___ Strongly disagree ___

16. E-mail would improve communication with my child's doctor

Strongly agree ___ Agree ___ Neutral ___ Disagree ___ Strongly disagree ___

17. E-mail would be a good/easy way to ask for an appointment

Strongly agree ___ Agree ___ Neutral ___ Disagree ___ Strongly disagree ___

18. I worry about hackers seeing my child's medical information

Strongly agree ___ Agree ___ Neutral ___ Disagree ___ Strongly disagree ___

I would feel comfortable discussing these things by e-mail with my child's doctor?

19 Ear ache Strongly agree ___ Agree ___ Neutral ___ Disagree ___ Strongly disagree ___

20 Cold symptoms Strongly agree ___ Agree ___ Neutral ___ Disagree ___ Strongly disagree ___

21 Fever Strongly agree ___ Agree ___ Neutral ___ Disagree ___ Strongly disagree ___

22 Diarrhea Strongly agree ___ Agree ___ Neutral ___ Disagree ___ Strongly disagree ___

23 Vomiting Strongly agree ___ Agree ___ Neutral ___ Disagree ___ Strongly disagree ___

24 Pink eye Strongly agree ___ Agree ___ Neutral ___ Disagree ___ Strongly disagree ___

25 Constipation Strongly agree ___ Agree ___ Neutral ___ Disagree ___ Strongly disagree ___

26 Feeding/Diet Strongly agree ___ Agree ___ Neutral ___ Disagree ___ Strongly disagree ___

This is a research study. Your participation is voluntary and will not affect the care your child receives today or in the future. Completing this survey will serve as your consent to participate in this study. Information gathered in this survey will be shared among the study team and may lead to a scientific article. Neither your name nor your child's name will appear on this survey.

27 **Weight** Strongly agree ___ Agree ___ Neutral ___ Disagree ___ Strongly disagree ___

28 **Immunizations** Strongly agree ___ Agree ___ Neutral ___ Disagree ___ Strongly disagree ___

29 **Sleep** Strongly agree ___ Agree ___ Neutral ___ Disagree ___ Strongly disagree ___

30 **Behavior** Strongly agree ___ Agree ___ Neutral ___ Disagree ___ Strongly disagree ___

31 **Colic** Strongly agree ___ Agree ___ Neutral ___ Disagree ___ Strongly disagree ___

32 **Development** Strongly agree ___ Agree ___ Neutral ___ Disagree ___ Strongly disagree ___

33 **Safety topics** Strongly agree ___ Agree ___ Neutral ___ Disagree ___ Strongly disagree ___

34 **Toilet training** Strongly agree ___ Agree ___ Neutral ___ Disagree ___

Strongly disagree ___

35 What would be your preferred way of receiving test and x ray results?

E-mail ___ Phone ___ Regular mail ___ Text message ___

Thank you for completing this questionnaire

Please hand form in to the front desk
